# Garlic-derived bioactive components in neuropathic pain: molecular mechanisms and therapeutic promise

**DOI:** 10.3389/fnmol.2026.1886063

**Published:** 2026-09-23

**Authors:** Asif Ali, Sorimuthu Revathi, Koilmani Emmanuvel Rajan, Chin Kun Wang, Subramanian Thangaleela

**Affiliations:** 1 Department of Nutrition, Chung Shan Medical University, Taichung, Taiwan; 2 Department of Emergency Medicine, Saveetha Medical College and Hospital, Saveetha Institute of Medical and Technical Science, Thandalam, India; 3 Behavioural Neuroscience Laboratory, Department of Animal Science, Bharathidasan University, Tiruchirappalli, India

**Keywords:** garlic bioactive compounds, mitochondrial dysregulation, neuropathic pain, organosulfur compounds, oxidative stress, health

## Abstract

Neuropathic pain is a chronic condition associated with maladaptive changes in nociceptive pathways, driven by oxidative stress, mitochondrial impairment, neuroinflammation, ion channel dysfunction, and apoptotic dysregulation. Current pharmacological therapies often provide limited relief but with adverse effects highlighting the need for more safer therapeutic approaches. Garlic (*Allium sativum*) and its bioactive organosulfur compounds including allicin, S-allyl cysteine, diallyl disulfide, diallyl trisulfide have gained increasing attention for their neuroprotective potential. This review summarizes the mechanistic role of garlic-derived compounds in neuropathic pain modulation, focusing on their antioxidant, anti-inflammatory, anti-apoptotic, and mitochondrial-stabilizing properties. These bioactives regulate key signaling pathways such as NF-κB, Nrf2/HO-1, PI3K/Akt, and JNK, while also modulating TRPV1 and TRPA1 ion channels involved in nociception and central sensitization furthermore, garlic compounds enhance endogenous antioxidant defense, preserve mitochondrial bioenergetics, and inhibit caspase-mediated neuronal apoptosis, thereby contributing to neuroprotection. Despite promising preclinical evidence, challenges such as variability in garlic preparations, limited clinical studies, and insufficient pharmacokinetic data remain. Overall, garlic-derived compounds represent a promising complementary strategy for neuropathic pain management, warranting further standardized clinical investigations to validate their therapeutic efficacy and safety.

## Graphical abstract

**Figure:**
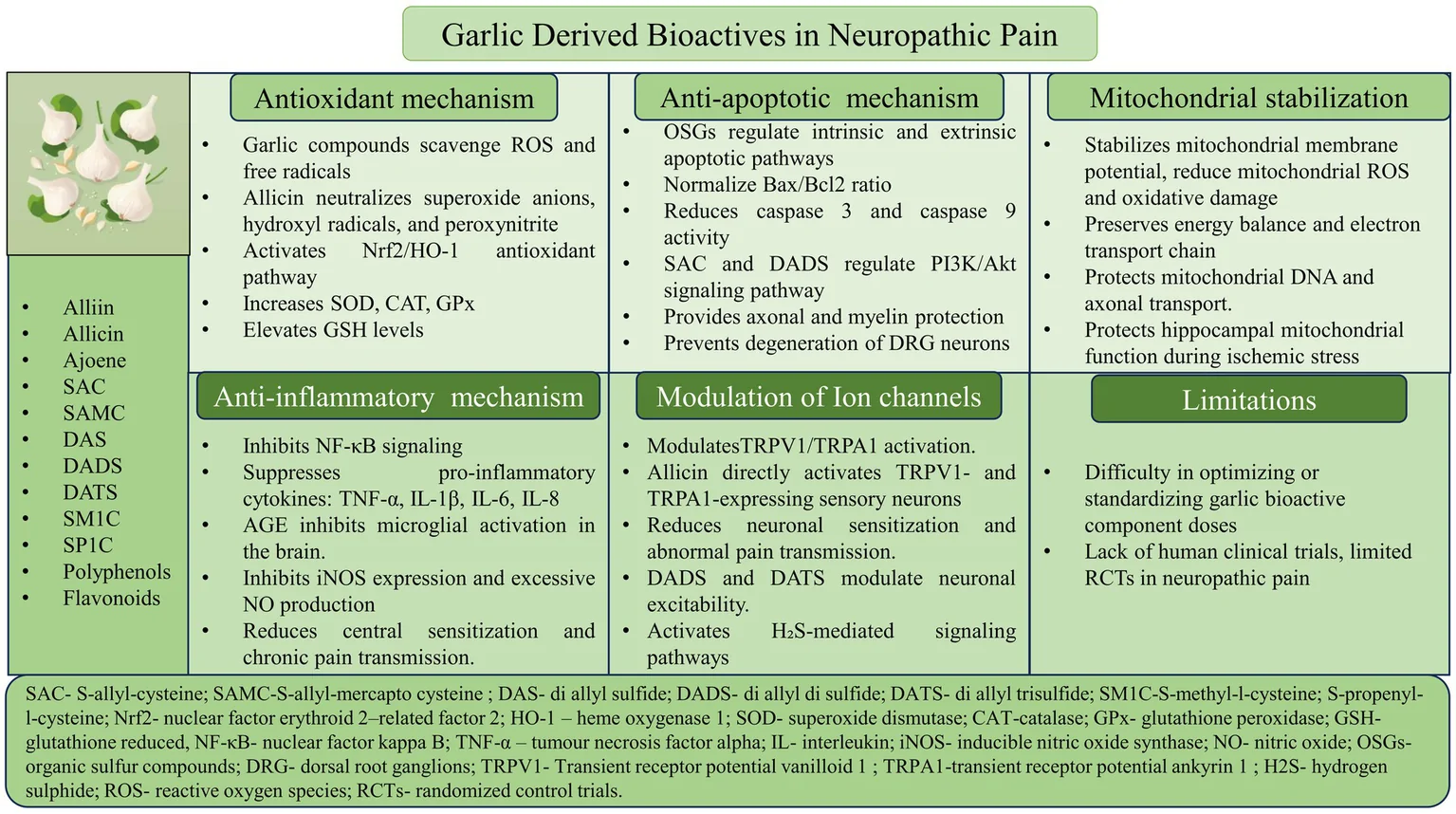

## 1 Introduction

Neuropathic pain (NP) is pain caused by a lesion or disease of the somatosensory nervous system and may involve either the peripheral or central somatosensory nervous system. Neuropathic pain includes various conditions like polyneuropathy, postherpetic neuralgia, trigeminal neuralgia, post-stroke pain (Baron et al., 2010). The earlier International Association for the Study of Pain (IASP) definition described neuropathic pain as pain initiated or caused by a primary lesion or dysfunction of the nervous system (Finnerup et al., 2016). However, the term “dysfunction” was considered insufficiently specific for clinical diagnosis, leading to a revised definition that emphasizes a lesion or disease affecting the somatosensory nervous system (Freynhagen et al., 2019). In contrast, nociceptive pain arises from actual or threatened damage to non-neural tissue and results from activation of nociceptors. It often diminishes as the underlying tissue injury or inflammation resolves (Wang et al., 2020). NP may be continuous or episodic and may occur spontaneously or in response to normally innocuous or painful NP pain can arise from diverse diseases or injuries affecting the peripheral or central somatosensory nervous system. Common etiologies include metabolic disorders such as diabetes, neurodegenerative and neurological disorders, vascular lesions, autoimmune diseases, cancer, trauma, infections, exposure to neurotoxic substances, and hereditary disorders. When no underlying cause can be identified despite appropriate clinical evaluation, the condition may be classified as idiopathic neuropathy (Colloca et al., 2017). NP is often characterized by burning, shooting, stabbing, or electric shock-like pain and may be accompanied by positive sensory symptoms such as paresthesia, dysesthesia, and allodynia, as well as negative sensory symptoms such as numbness or sensory loss. These conditions are often seen associated with multiple sclerosis, Guillain-Barré syndrome, diabetes and few other neuronal disease conditions (Sic et al., 2024). Epidemiological studies have shown that NP is associated with substantial impairment in quality of life and remains underrecognized and inadequately managed. NP is estimated to affect approximately 7–10% of the general population, although prevalence estimates vary according to the population studied and the diagnostic criteria used (Bouhassira, 2019).

Unlike nociceptive pain, which is initiated by the activation of nociceptors in response to actual or threatened tissue damage, NP arises from pathological alterations within the somatosensory nervous system. These alterations can lead to abnormal spontaneous or evoked neuronal activity, including ectopic firing, peripheral and central sensitization, and impaired inhibitory signaling than from activation of nociceptors by non-neural tissue damage. Comparing to the other pains in tissues or muscle, which often heals over time, NP is hard to subside and cause worst outcome, leading to chronic pain. In many conditions it involves both nerve related and tissue related pain mechanisms. Although neuropathic and nociceptive pain are mechanistically distinct categories, they can coexist within the same clinical condition, giving rise to mixed pain phenotypes. Thus, pain mechanisms are better understood as overlapping rather than mutually exclusive in many clinical settings (Cohen and Mao, 2014). The International Association for the Study of Pain (IASP), in collaboration with an interdisciplinary task force of pain specialists, developed a classification of chronic neuropathic pain within the 11th revision of the International Classification of Diseases (ICD-11). Chronic neuropathic pain is classified as a form of chronic secondary pain and is further categorized into peripheral and central neuropathic pain. Peripheral neuropathic pain includes painful polyneuropathy, postherpetic neuralgia, painful radiculopathy, trigeminal neuralgia, and pain following peripheral nerve injury, whereas central neuropathic pain includes pain associated with spinal cord injury, brain injury, stroke, and multiple sclerosis (Scholz et al., 2019).

NP remains challenging to treat effectively across etiologies because its pathophysiology is heterogenous. This fundamental difficulty underpins the limited and partial efficacy of current treatment therapies. Current pharmacological treatments primarily provide symptomatic relief and show substantial inter-individual variability in efficacy and tolerability. So that multimodal, mechanism-guided approaches are necessary (Moulin et al., 2014; Helfert et al., 2015; Scholz et al., 2019). First-line pharmacological therapies commonly include antidepressants, particularly SNRIs and tricyclic antidepressants, and α2δ calcium-channel ligands such as gabapentin and pregabalin, although treatment selection depends on etiology, clinical phenotype, comorbidities, and individual response. However, overall responses are variable and many patients require multidisciplinary, multimodal strategies, including nonpharmacologic and interventional approaches, tailored to the patient’s specific mechanism and sensory profile (Moulin et al., 2014; Gilron et al., 2015).

The multifactorial conditions involving neuroinflammation, oxidative stress and aberrant neuronal signaling often limits the efficacy of conventional pharmacological therapies. These limitations have stimulated interest in plant-derived bioactive compounds as potential sources of novel therapeutic candidates for NP. A wide range of medicinal plants including *Crocus sativus* (saffron), *Citrullus colocynthis* (bitter apple), *Curcuma longa* (turmeric), *Lavandula angustifolia* (lavender), *Cannabis sativa* (Olalekan et al., 2024), *Acorus calamus, Artemisia dracunculus, Butea monosperma, Citrullus colocynthis, Curcuma longa, Crocus sativus, Elaeagnus angustifolia, Ginkgo biloba, Mitragyna speciosa, Momordica charantia, Nigella sativa, Ocimum sanctum, Phyllanthus amarus*, *Pterodon pubescens Benth*, *Rubia cordifolia* and *Salvia officinalis* have demonstrated protective effects against NP in both experimental and clinical studies (Forouzanfar and Hosseinzadeh, 2018). Bioactive constituents derived from these plants have demonstrated potential effects against neuropathic pain through multiple mechanisms, including anti-inflammatory, antioxidant, anti-apoptotic, neuroprotective, and modulation of voltage-gated calcium channels. Among these phytochemicals, sulfur-containing organosulfur compounds derived from garlic, including diallyl disulfide (DADS) and diallyl trisulfide (DATS), have demonstrated promising pharmacological activities relevant to pain management, neuroprotection, and NP (Olalekan et al., 2024). In this review, we discuss the potential of garlic-derived bioactive compounds in the modulation of neuropathic pain, with particular emphasis on their antioxidant, anti-inflammatory, neuroprotective, and neuronal signaling mechanisms.

## 2 Literature search strategy

A literature search was performed using PubMed, Scopus, Web of Science, and Google Scholar using combinations of the terms: “*Allium sativum*,” garlic, allicin, S-allyl cysteine, diallyl disulfide, diallyl trisulfide, “neuropathic pain,” “peripheral neuropathy,” “diabetic neuropathy,” “chemotherapy-induced peripheral neuropathy,” neuroinflammation, Nrf2, HO-1, NF-κB, PI3K/Akt, TRPV1, TRPA1, mitochondrial dysfunction, and Drp1. Articles were screened by title/abstract, followed by full-text review where relevant. Priority was given to studies directly addressing NP or nerve-injury models. Studies conducted in non-neuropathic neurological models, including neurodegenerative, ischemic, and traumatic neural injury models, were retained when they provided mechanistic evidence relevant to pathways implicated in NP; however, these studies were explicitly interpreted as supportive rather than direct evidence of efficacy in NP. Human studies were considered where available, with particular attention to the limited clinical evidence directly evaluating garlic-derived bioactives in NP. Reference lists of relevant articles were also examined for additional relevant studies.

## 3 Garlic and its bioactive compounds

Garlic (*Allium sativum* L.) is a globally cultivated food and medicinal plant that contains a wide range of bioactive compounds with potential health-promoting and therapeutic properties Garlic has traditionally been consumed as a food and used as a health-promoting ingredient across different cultures. The health benefits attributed to garlic are associated with a diverse range of phytochemicals, particularly organosulfur compounds (OSCs) (Rajpoot and Sharma, 2026). Garlic contains diverse OSCs, including cysteine sulfoxides such as alliin, thiosulfinates such as allicin, lipid-soluble sulfides and polysulfides such as diallyl disulfide (DADS) and diallyl trisulfide (DATS), and water-soluble derivatives such as S-allyl cysteine (SAC) and S-allyl mercapto cysteine (SAMC) (Hu et al., 2021). Aqueous and alcohol based garlic extracts contain water soluble OSCs SAC, SAMC, S-methyl-l-cysteine (SM1C), and S-propenyl-l-cysteine (SP1C) (Rajpoot and Sharma, 2026). In addition to OSCs, garlic contains other phytochemical classes, including polysaccharides, saponins, phenolic compounds, tannins, and volatile constituents such as linalool and geraniol, which have also been investigated for various biological activities. *In vitro* and *in vivo* studies have showed that garlic-derived OSCs exhibit diverse biological activities including anti-inflammatory, anti-oxidative, anti-diabetic, anti-microbial, anti-aging, anti-cancer activities as well as cardioprotective effects and immunomodulatory properties (El-Saadony et al., 2024).

Garlic-derived preparations, including garlic oil macerates, essential oils, garlic powder, aged garlic extract (AGE), and black garlic (BG), have been investigated for their potential health-promoting and therapeutic effects (Imaizumi et al., 2023). AGE is produced by prolonged extraction of raw garlic, typically in an aqueous ethanol solution, under controlled conditions, resulting in an odorless preparation enriched in SAC and SAMC. Lipid-soluble compounds in AGE include DAS, triallyl sulfide, DADS, diallyl polysulfides shows antioxidant effects (Awazu and Horie, 2008). BG was processed from fresh garlic under controlled thermal and moisture conditions with or without additives which was ready to eat, with sweet taste and lesser pungent odor. Black garlic is produced by heating fresh garlic under controlled temperature and humidity conditions for a prolonged period, typically at approximately 40–90 °C and 60–90% relative humidity, although processing conditions vary among studies and commercial products (Jing, 2020). During the controlled thermal aging of garlic, Maillard reactions and related chemical transformations occur, resulting in changes in color, flavor, texture, and phytochemical composition. During the later stages of the Maillard reaction, high-molecular-weight nitrogen-containing compounds known as melanoidins are formed, contributing to the characteristic dark color and antioxidant properties of BG (Yuan et al., 2018). During thermal aging, unstable compounds such as allicin are substantially degraded, while relatively stable water-soluble sulfur compounds, particularly SAC and SAMC, may accumulate depending on the processing conditions (Ahmed and Wang, 2021). Studies of black garlic from different cultivars and geographical origins have detected phenolic compounds such as chlorogenic acid, gallic acid, benzoic acid, and vanillic acid, although their profiles and concentrations vary according to processing and raw-material characteristics (Eyupoglu, 2019).

Cysteine sulfoxides, particularly alliin, serve as important precursors of garlic thiosulfinates, most notably allicin (Verma et al., 2023). When garlic cloves are crushed or mechanically disrupted, the enzyme alliinase, which is compartmentalized in the vacuole, comes into contact with cysteine sulfoxides such as alliin. Allicin is relatively unstable and undergoes spontaneous decomposition to form various secondary sulfur-containing compounds, including diallyl disulfide (DADS), diallyl trisulfide (DATS), and other sulfides and polysulfides (Amagase, 2006). Allicin is highly reactive and can interact with thiol-containing proteins, peptides, and low-molecular-weight thiols, leading to the formation of S-allylmercapto adducts. Owing to its chemical instability and rapid reactivity, intact allicin is not readily detected in the systemic circulation following oral consumption of raw garlic (Lawson and Wang, 2005). Garlic is consumed in fresh and processed forms and provides various nutrients, including carbohydrates, proteins, dietary fiber, vitamins, minerals and trace elements (Subramanian et al., 2020).

According to Vecchi et al. (2025), fresh garlic comprises of 26–30% carbohydrates with 1.5% dietary fiber, 1.5–2.1% proteins, 0.1–0.2% lipids, 1.1–3.5% sulfur compounds, 17.16–42.53 mg gallic acid equivalents, 0.04–0.11% saponins, 0.015% vitamins, 0.7% minerals such as vitamin B group, C and E, other elements like sodium, calcium, potassium, phosphorus, magnesium, zinc, copper, iron, manganese and selenium (Vecchi et al., 2025). Garlic contains enzymes myrosinase, allinase and peroxidase, polyphenolic compounds including, gallic acid, rutin, quercetin, ferulic acid, p-coumaric acid, naringenin, apigenin, isorhamnetin, apigenine, kaempferol-3-O-glucoside-7-O-alpha-1-rhamnoside, kaempferol 3,7-di-O-rhamnoside, luteoline and kaempferol-3-O-glucoside and 17 amino acids (Dziri et al., 2012; Yamaguchi et al., 2021). Fresh garlic is rich in γ-glutamylcysteine, which participates in the biosynthetic pathway leading to the cysteine sulfoxide alliin. Garlic contains more than 20 different OSCs, classified as water-soluble and oil-soluble sulfur compounds. Alliin, SAC and SAMC are water-soluble, Allicin, DAS, DADS, DATS are oil-soluble compounds (Subramanian et al., 2020). SAC produced from γ-glutamyl-S-allyl-L-cysteine via an enzymatic transformation with the help of γ-glutamyltranspeptidase. Among other OSCs, SAC is the major compound, in aqueous garlic extract which demonstrated high biological activity, bio availability and effective properties against various diseases (Patiño-Morales et al., 2021), and neuroprotective activities in rat ischemia model (El-Saber Batiha et al., 2020). SAC inhibit Nuclear Factor-kB (NF-κB) activation in T cells and possess anti-inflammatory activities by inhibiting nitric oxide synthase expression. SAC exhibit anti-oxidant activity and acts as free radical scavenger on rat ischemia model (Subramanian et al., 2020).

During garlic aging, SAMC can be formed through pathways involving γ-glutamyl-S-allyl mercapto cysteine (GSAMC), a putative precursor of SAMC. Endogenous γ-glutamyl transpeptidase activity contributes to the conversion of GSAMC to SAMC, while allicin may participate in the formation of GSAMC (Fujii et al., 2018). Allicin is highly sensitive to light and heat, hence easily metabolized into various compounds such as DAS, DADS, DATS. The hydrophobic nature of allicin makes it easily absorbed into the cell membrane and metabolized rapidly to exert its pharmacological effects (Chan et al., 2013). DAS is lipophilic, derived from oxidized allicin during crushing of garlic. DADS is a major lipid-soluble organosulfur compound found in garlic oil and has been reported to modulate cell-cycle and apoptotic pathways, including pathways involving p53 and cyclin B1, in experimental cancer models (Altonsy and Andrews, 2011). Studies about the bioavailability and bioaccessibility of garlic bioactive compounds revealed that, water-soluble organo-sulfur compounds can be detected in the plasma, liver and kidney, but the oil-soluble organo-sulfur compounds such as allicin, ajoene, vinyldithiins are not detected in the blood or urine (Tedeschi et al., 2022). Thus, the water-soluble garlic compounds are rapidly absorbed in the gastrointestinal tract, has prominent anti-oxidant properties.

## 4 Neuropathic pain mechanisms relevant to garlic-derived bioactive compounds

NP encompasses a heterogeneous group of conditions with diverse etiologies and clinical manifestations, involving complex interactions among neuronal, immune, mitochondrial, and molecular mechanisms. A comprehensive description of the classification and pathophysiology of all NP conditions is beyond the scope of this review. Therefore, this section provides a focused overview of selected pathophysiological mechanisms that are particularly relevant to the potential therapeutic actions of garlic-derived bioactive compounds, including mitochondrial dysfunction and oxidative stress, neuroimmune signaling, ion-channel dysregulation, and TRPV1/NMDA receptor-associated pathways. These mechanisms are discussed in the context of available evidence linking garlic-derived constituents with the modulation of NP-related molecular and cellular processes.

### 4.1 Mitochondrial dysfunction, Drp1, and ROS signaling

Growing evidence suggests that mitochondrial dysfunction is a major contributing factor in the initiation and maintenance of NP (Trecarichi et al., 2022). Under normal conditions, mitochondria serve as energy supply center of cells and play a crucial role in maintaining neuronal homeostasis (Willemen et al., 2023). In response to stressful stimuli, neurons require more ATP and oxygen, which may disrupt cellular metabolism and lead to excessive production of ROS and reactive nitrogen species (RNS) (Li et al., 2025). Mitochondrial dysfunction can reduce cellular energy production, which leads to a cascade of events that promote neuronal hyper-excitability and abnormal pain signaling, such as disruption of ion pump function (Na^+^/K^+^-ATPase), dysregulation of Ca^2+^ signaling, release damage-associated molecular patterns (DAMPs), including mitochondrial DNA (mtDNA), ROS, and cytochrome c, into the extracellular space (Trecarichi et al., 2022).

Studies aiming to figure out the possible involvement of dynamin-related protein 1 (Drp1)-mediated mitochondrial fragmentation, leads to ROS generation thereby implicated the pathogenesis of NP (Dai et al., 2020). Mitochondria are important organelles in the cells particularly in neurons. Mitochondria are highly dynamic organelles that continuously undergo fusion and fission to maintain mitochondrial quality, distribution, and function, particularly in energy-demanding neurons (Otera et al., 2013). Drp1 is a dynamin-related GTPase that regulates mitochondrial fission by assembling at mitochondrial constriction sites (Tsushima et al., 2018), mitochondrial fission, and mitophagy (Horbay and Bilyy, 2016). Any mutation in Drp1 gene or abnormal expression cause mitochondrial diseases. Drp1 induced mitophagy have been reported in neurodegenrative diseases, ischemia and traumatic brain injury (Wu et al., 2017). In mitochondrial ROS are generated as metabolic products as a result of oxidative phosphorylation. These ROS plays a critical role in the signaling pathways, a balance between ROS production and elimination is therefore becomes vital in maintaining the redox balance. Dysregulation of mitochondrial dynamics, including aberrant Drp1 activity, can disturb mitochondrial homeostasis and contribute to oxidative stress and neuropathic pain. However, the effects of Drp1 modulation appear to depend on the cellular compartment, disease model, and degree of mitochondrial fission (Gibellini et al., 2015). Conversely, loss or inhibition of Drp1 can impair mitochondrial fission and promote excessive mitochondrial elongation, indicating that both excessive and insufficient fission may disrupt mitochondrial homeostasis depending on the cellular context (Cho et al., 2013).

Though the specific mechanism beneath the mitochondria-ROS-NP still remains unclear, researchers doubt the possible relation between Drp1, ROS and NP. The widely accepted prominent pathophysiological mechanisms of NP are development of spontaneous or ectopic activity at the injury site, molecular changes occur in injured dorsal root ganglia due to loss of trophic support, Wallerian degeneration exposes nerves to cytokines and growth factors, partial denervation increases trophic factors causing nociceptor sensitization, peripheral sensitization, central sensitization, impaired inhibitory modulation and microglial activation (Gilron et al., 2015; Gierthmühlen and Baron, 2016). Drp1-mediated mitochondrial dysfunction and fragmentation induced the ROS production and plays an important role in NP. Post-translational modifications of Drp1 can regulate its recruitment to mitochondria and mitochondrial fission. Under pathological conditions, excessive Drp1 activation may contribute to mitochondrial fragmentation, oxidative stress, and mitochondrial dysfunction. Under pathological circumstances dysregulated Ca^2+^ homeostasis may exacerbate the Drp1–mitochondrial dysfunction–ROS cycle and contribute to the development and maintenance of NP (Dai et al., 2020). Evidence regarding the role of Drp1 in NP is complex and may depend on the tissue, disease model, and degree of Drp1 modulation. While inhibition of Drp1-mediated fission attenuated neuropathic pain in some peripheral nerve and chemotherapy-induced models, targeted Drp1 overexpression in the spinal dorsal horn was reported to alleviate pain hypersensitivity and reduce mitochondrial oxidative stress in a spared nerve injury model (Zhang et al., 2015). NP model studies revealed changes in Drp1 expression in dorsal root ganglia, which plays a crucial role in pain transmission. In experimental models of hyperglycemia-induced diabetic neuropathy, DRG neurons exhibit mitochondrial injury associated with altered mitochondrial dynamics and increased Drp1-related signaling (Leinninger et al., 2006; Edwards et al., 2010). Dendrites and axons of dorsal root ganglion neurons showed increased Drp1 expression, confirms the involvement of Drp1 in pain transmission, development and regulation (Amiri and Hollenbeck, 2008). Overexpression Drp1, restores mitochondrial morphology and alleviates NP by stabilizing the inner mitochondrial membrane (Zhang et al., 2022). Given the established contribution of mitochondrial dysfunction and oxidative stress to NP, modulation of these pathways may represent an important mechanism through which garlic-derived bioactive compounds exert their potential neuroprotective and analgesic effects.

### 4.2 Microglial contribution

Decades of neuroimmune research, have established that microglia respond to nerve injury and modulate NP states. Thus, interactions have emerged as important potential targets for attenuating abnormal neuronal activity and pain hypersensitivity in NP (Malcangio and Sideris-Lampretsas, 2025). Nerve injury can trigger microglial activation through signaling molecules, including neuregulin-1, matrix metalloproteinases (MMP-2 and MMP-9), chemokine (C-C motif) ligand 2 (CCL2), and fractalkine. These signaling pathways contribute to the recruitment and activation of microglia within the injured peripheral and central nervous systems. Activated microglia subsequently release pro-inflammatory mediators, including interleukin (IL)-6, IL-1β, and tumor necrosis factor-α (TNF-α), which can enhance neuronal excitability and disrupt inhibitory neurotransmission, thereby contributing to the development and maintenance of NP (Leoni et al., 2025). Under physiological conditions, specialized sensory neurons in peripheral tissues detect noxious stimuli and transmit nociceptive signals to the dorsal horn of the spinal cord. Following nerve injury, however, abnormal neuronal activity can activate microglia in the spinal dorsal horn, which subsequently release inflammatory mediators that modulate neuronal excitability and nociceptive signaling. Dysregulated microglia–neuron interactions can therefore contribute to the amplification and persistence of pathological pain signaling in NP.

NP is altered by the excitability of primary sensory neurons and dorsal horn neurons, accompanied by changes in inflammatory mediators like cytokines and chemokines. Microglia respond to neuronal activity through multiple signaling pathways, including purinergic and neurotransmitter-mediated signaling. Microglia can sense extracellular neurotransmitters and neuromodulators through receptors such as GABA-B, glutamate, β2-adrenergic, and 5-HT2B receptors, thereby allowing them to monitor changes in neuronal activity (Malcangio and Sideris-Lampretsas, 2025). In addition to neurotransmitter signaling, microglia rapidly respond to changes in the extracellular ionic environment, particularly extracellular K^+^ concentrations, which are closely coupled to neuronal activity (Rasmussen et al., 2020). The tandem pore domain halothane-inhibited K^+^ channel 1 (THIK1) contributes to the maintenance of the hyperpolarized resting membrane potential of microglia, which is important for its motility and surveillance of the surrounding environment. Elevated extracellular K^+^ can depolarise microglia and promote THIK-1 dependent process, therby reducing the microglial activities (Madry et al., 2018). uch alterations in extracellular ionic homeostasis may contribute to abnormal microglial responses and potentially influence microglia–neuron interactions during NP.

Following peripheral nerve injury, increased extracellular ATP signaling in the spinal dorsal horn contributes to nociceptive processing and microglial activation. ATP can enhance excitatory neurotransmission by increasing presynaptic glutamate release and promoting excitatory responses in spinal projection neurons (Gu and MacDermott, 1997). In addition, extracellular ATP activates microglia through purinergic receptors and contributes to the development and maintenance of neuropathic pain. ATP signaling in the spinal cord can involve both neuronal and glial sources, including astrocytes, which release ATP through regulated release mechanisms and thereby influence microglial activity (Inoue, 2022). Following nerve injury, transcriptional regulators such as interferon regulatory factors and MafB contribute to changes in microglial gene expression including the regulation of purinergic signaling pathways (Masuda et al., 2012; Tozaki-Saitoh et al., 2019). These findings highlight the importance of purinergic signaling in the microglial response to neuropathic conditions.

Among purinergic receptors, P2X4R has been particularly implicated in microglia-mediated neuropathic pain. Pharmacological or genetic inhibition of P2X4 signaling has been reported to reduce neuropathic pain hypersensitivity (Ulmann et al., 2008). Activation of P2X4 receptors in microglia triggers the release of brain-derived neurotrophic factor (BDNF), BDNF subsequently acts on tropomyosin receptor kinase B (TrkB) receptors on dorsal horn neurons, leading to downregulation of the neuronal K^+^–Cl^−^ cotransporter KCC2. This disrupts the chloride balance, reverses inhibitory GABA and glycine signaling, and leads to neuronal disinhibition and increased pain transmission (Coull et al., 2005; Beggs et al., 2012). Thus, P2X4R dependent microglial signaling provides an important link between extracellular ATP, microglial activation, altered inhibitory neurotransmission, and NP (Ulmann et al., 2008). The involvement of microglial activation and neuroinflammatory signaling in NP provides a potential mechanistic basis for investigating the anti-inflammatory effects of garlic-derived bioactive compounds.

### 4.3 Transient receptor potential vanilloid 1 (TRPV1) and N-methyl-D-aspartate (NMDA) receptors

Emerging evidence suggests that transient receptor potential vanilloid 1 (TRPV1) and N-methyl-D-aspartate (NMDA) receptors along with voltage-gated of sodium (Naᵥ), calcium, (Caᵥ), and potassium (Kᵥ) channels play important role in pain signaling pathways (Kang et al., 2021). Dysregulation or reduced in Naᵥ, Caᵥ, and Kᵥ channel activity, can disrupt neuronal excitability and contribute to the development and maintenance of NP (Madry et al., 2018; Goodwin et al., 2020). TRPV1 is a non-selective cation channel predominantly expressed in peripheral sensory neurons, where it is activated by thermal and inflammatory response to various noxious stimuli, leading to nociceptive signal initiation. The TRP channel family comprises nonselective cation channels involved in diverse physiological processes, including pain perception and inflammation (Legrand et al., 2020). Among these, transient receptor potential ankyrin 1 (TRPA1) is a calcium-permeable channel expressed in nociceptive neurons of the dorsal root and trigeminal ganglia, where it contributes to pain signaling (Earley, 2012).

N-methyl-D-aspartate (NMDA) receptors are ionotropic glutamate receptors expressed throughout the CNS and play a crucial role in synaptic plasticity, which is essential for physiological processes as well as pathological conditions including chronic pain (Chen et al., 2014; Kang et al., 2021). Activation of NMDA receptors induces Ca^2+^ influx, which activates Ca^2+^/calmodulin-dependent protein kinase II (CaMKII) via calmodulin. Activated CaMKII enhances excitatory synaptic transmission, in part by increasing the phosphorylation, trafficking, and functional activity of α-amino-3-hydroxy-5-methyl-4-isoxazolepropionic acid (AMPA) receptors. Furthermore, interactions between CaMKII and the GluN2B (NR2B) subunit of the NMDA receptor can facilitate sustained kinase activity and prolonged excitatory signaling. Collectively, these molecular events contribute to persistent synaptic potentiation, central sensitization, and the development and maintenance of chronic pain states (Ian et al., 2025). NMDA receptors also functionally interact with TRPV1 channels in sensory neurons, including those in the trigeminal ganglia, contributing to mechanical hyperalgesia (Lee et al., 2018). NMDA receptor activation promotes phosphorylation and sensitization of TRPV1 via CaMKII- and protein kinase C (PKC)-dependent pathways. Pharmacological inhibition of TRPV1 or these kinases attenuates NMDA-induced nociceptive responses, highlighting the role of TRPV1 in pain signaling (Mandlem et al., 2024). Dysregulation of TRPV1, NMDA receptors, and voltage-gated ion channels contributes to neuronal hyperexcitability and pain transmission, modulation of these pathways may represent another mechanism through which garlic-derived constituents could influence NP.

## 5 Evidence for garlic-derived compounds in neuropathic pain models

Garlic is one of the most promising medicinal plants for treating various chronic diseases in humans, including knee osteoarthritis, rheumatoid arthritis, and peripheral arterial occlusive disease (stage II) (Hernández-Cruz et al., 2022). Common garlic preparations include garlic powder (dried garlic), steam-distilled garlic oil, garlic oil macerate, and AGE. Garlic oil is a rich source of OSCs, which have been shown to attenuate NP in chronic constriction injury (CCI) rat models (Wang et al., 2020). OSCs such as allicin and DADS have been extensively evaluated in preclinical models and some clinical studies for their analgesic potential (Bautista et al., 2005; Hernández-Cruz et al., 2022). Previous studies have demonstrated that DADS and DATS alleviate CCI-induced NP in rats by restoring hydrogen sulfide (H_2_S), BDNF, and nuclear factor erythroid 2–related factor 2 (Nrf2) levels in the sciatic nerve and dorsal root ganglia (Wang et al., 2020). Additionally, OSCs inhibit the synthesis of prostaglandins, pro-inflammatory cytokines, and interferon-γ by reducing cyclooxygenase-2 (COX-2) activity while enhancing anti-inflammatory cytokine levels, thereby contributing to pain reduction (Hernández-Cruz et al., 2022). The effects of garlic and its major bioactive constituents on NP–related and supportive mechanistic pathways are summarized in Table 1.

**Table 1:** Summary of preclinical and experimental studies evaluating the effects of garlic and its bioactive constituents.

Effect of garlic and its compounds		
Bioactive components	Study evaluation	References
Ethanol (95%) extract of garlic	Administration of a 95% ethanolic garlic extract (45 mg/kg body weight/day for 28 days) significantly reduced serum glucose levels and nociceptive responses in tail-flick, hot plate, allodynia, and formalin tests in alloxan-induced diabetic mice.	Kumar and Reddy (1999)
Diallyl disulfide	14 days administration of diallyl disulfide (25 and 50 mg/kg) restored H₂S, BDNF, and Nrf2 levels.	Wang et al. (2020)
Diallyl trisulfide	14 days administration of (20 and 40 mg/kg) diallyl trisulfide activate H_2_S-BDNF-Nrf2 signaling pathway	Wang et al. (2020)
Diallyl trisulfide	DATS (dissolved in corn oil, 40 mg/kg/day) was orally administered for 3 days before SCII and the last dose was given 10 min before reperfusion activated AMPK, protected mitochondrial damage and reduced oxidative stress recovered the neuronal damage in SCII rats.	Sun et al. (2023)
S-allyl cysteine (SAC)	15 days administered of SAC (100 mg/kg, i.p.) enhanced neurotrophin levels, NGF (cortex), BDNF (cortex and striatum), VEGF (striatum), pTrkB, pAKT, and pERK (cortex and striatum).	Bautista-Perez et al. (2026)
S-allyl cysteine (SAC)	SAC supplementation suppressed the increase in pro-inflammation levels (TNF-α/IL1/IL8) caused by 6-OHDA (*p* < 0.05) in SH-SY5Y cells injured by 6-OHDA	Yeni et al. (2024)
Allicin	5 or 10 mg/kg allicin increased the protein levels of HSP70, increased the phosphorylation of Akt, reduced the iNOS protein expression, reduced the levels of ROS and enhanced the NADH levels in traumatic spinal cord injured mice	Wang and Ren (2016)
Allicin	Allicin treatment significantly attenuated glutamate-induced toxicity by HSP70/iNOS pathway-mediated inhibition of oxidative stress.	Liu et al. (2015)
Allicin	Pretreated with allicin (1, 10 and 50 mg/kg) for 2 weeks, significantly suppressed the accumulations of protein and lipid peroxidation products, and increased the activities of CAT, SOD, GPX and GST in infrarenal aortic occlusion-induced spinal cord I/R injury.	Liu et al. (2015)
Allicin	50 mg/kg of allicin via intraperitoneal injection for 14 consecutive days, improve the motor dysfunction and dopaminergic neuron loss in PD mice. Allicin activate the PKA/p-CREB/BDNF signaling pathway and increase the DAT level in brain tissue, regulate the expression of BAX and Bcl-2, and reduce neuronal apoptosis	Zeng et al. (2025)
Ajoene	allicin derivative ajoene enhance the activation of the transient receptor potential channel TRPA1	Yassaka et al. (2010)
*Z*-ajoenes	25 mg/kg orally administered *Z*-ajoenes 30 min prior to the induction of transient forebrain ischemia by occlusion of the common carotid arteries for 5 min showed neuroprotective effect. In addition, *Z*-ajoene significantly decreased lipid peroxidation.	Yoo et al. (2014)
AGE	250 mg kg − 1 per day of AGE diluted in tap water orally by gavage for 15 days prior to the trauma demonstrated decreased MDA levels and increased SOD levels when compared with the SCI group	Cemil et al. (2012)
ABGE	30 and 60 min after the acute administration of the extract at different doses orally administered with ABGE (0.03–1 g kg^−1^; 10 mL kg^−1^) dose-dependently relieved post-inflammatory visceral pain induced by (DNBS) injection in rats. ABGE suppressed LPS-induced gene expression of IL-1β, IL-6, NF-kB, and TNF-α.	Libero et al. (2024)

AGE, aged garlic extract; ABGE, aged black garlic extract; AMPK, adenosine monophosphate activated protein kinase; CAT, catalase; DATS, Diallyl trisulfide; SOD, superoxide dismutase; MDA, malondialdehyde; SCI, spinal cord injury; LPS, lipopolysaccharide; DNBS-2,4, di-nitrobenzene sulfonic acid; TRPA1, transient receptor potential ankyrin 1; PKA, protein kinase A; p-CREB, phosphorylated cyclic dependent response element binding protein; BDNF-brain derived neurotrophic factor; GPX, glutathione peroxidase; GST-glutathione S-transferase; PD, parkinson’s disease; 6-OHDA, 6-Hydroxydopamine; TNF-α, tumor necrosis factor alpha; IL, interleukin; NGF, neuronal grrowth factor; VEGF-vascular endothelial growth factor; pTrkB, phosphorylated tyrosine kinase receptor B; pAKT, phosphorylated AKT (Protein Kinase B); pERK, phosphorylated extracellular regulated kinase; SCII, Spinal cord ischemia–reperfusion injury; ROS-reactive oxygen species; NADH, nicotinamide adenyl dineucleotide, reduced form.

Allicin is an important bioactive substance in garlic, exerts a broad spectrum of pharmacological activities and is considered to have therapeutic potential in many neurological disorders (Liu et al., 2015). Allicin treatment preserved the function of mitochondrial respiratory chain complexes and inhibited the production of ROS and the release of mitochondrial cytochrome c in the spinal cord ischemia–reperfusion injury rabbit model (Zhu et al., 2012). Thiosulfinates and isothiocyanate compounds contain reactive electrophilic chemical groups that react with cysteine residues within the TRPA1 channel N terminus, leading to channel activation. Ajoene also contains reactive electrophilic chemical groups likely to target TRPA1 channel (Yassaka et al., 2010).

Intriguingly, allicin and DADS can activate TRPA1 in sensory neurons and induce vasodilation through activation of capsaicin-sensitive perivascular sensory nerve endings. Both compounds have been reported to activate cloned TRPA1 channels (Bautista-Perez et al., 2026). TRPA1 is an important sensor and contributes to neuroimmune signaling and trigger the release of neuropeptides, which may contribute to antioxidant and anti-inflammatory effects (Viana, 2018). In addition, DADS and DATS have been reported to attenuate neuropathic pain in rats subjected to chronic constriction injury (CCI), potentially through modulation of the H₂S–BDNF–Nrf2 signaling pathway (Wang et al., 2020). Beyond NP, garlic-derived organic polysulfides havedemostrated neuroprotective effects following post-Ischemia–reperfusion injury, including improvement in nerve function, reduction of infarct size, attenuation of inflammatory responses, and inhibition of cellular apoptosis (Wang et al., 2025). Another garlic-derived bioactive compound SAC has shown neuroprotective properties. SAC promote post-ischemic recovery, improvr motor performance and preserve long-term recognition memory, effects that may be associated with increased cell proliferation, neurotrophin levels (Bautista-Perez et al., 2026).

AGE has potent antioxidant properties and has demonstrated neuroprotective effects, particularly in experimental models of cerebral ischemia (Cemil et al., 2012). In ischemia–reperfusion (I/R) models, AGE treatment has been reported to enhance antioxidant defense while reducing pro-inflammatory cytokine production and caspase-3 activation, thereby attenuating neuronal injury (Cemil et al., 2012). AGE and its bioactive components modulate multiple signaling pathways associated with oxidative stress and neuroinflammation, contributing to neuroprotection in neurodegenerative disorders such as Alzheimer’s, Huntington’s, and Parkinson’s diseases (Qu et al., 2016; Farooqui and Farooqui, 2018). Furthermore, aged black garlic extract (ABGE) has demonstrated analgesic potential in an experimental model of post-inflammatory visceral pain. In a DNBS-induced rat model, acute administration of ABGE significantly attenuated pain-related behavioral responses. A higher dose of ABGE (1 g/kg) markedly reduced nociceptive behavioral responses and the frequency of abdominal contractions induced by colorectal distension (Libero et al., 2024). Although this model represents post-inflammatory visceral pain rather than neuropathic pain, these findings provide preliminary evidence supporting the analgesic potential of black garlic-derived preparations.

Ethanolic fermented garlic extract reported to prevent monosodium glutamate (MSG) induced working memory impairment in adolescent male Wistar rats (Nurmasitoh et al., 2018). In addition, linalool identified in ethanol extracts of black garlic has demonstrated neurite outgrowth-promoting activity in neuronal cultures. In particular, treatment with, of black garlic has demonstrated neurite outgrowth-promoting activity in neuronal cultures. In particular, treatment with black garlic ethanol extract at 60 μg/mL significantly stimulated neurite outgrowth in rat primary hippocampal neurons and promoted axonal and dendritic growth, as well as the formation of functional synapses (Munni et al., 2023). S-allyl cysteine (SAC), another major water-soluble organosulfur compound derived from garlic, has also demonstrated neuroprotective and neurotrophic properties, including potential benefits in learning and memory impairment. SAC has been reported to exert antioxidant activity and ameliorate neuronal dysfunction associated with cognitive impairment. Furthermore, pretreatment with SAC protected SH-SY5Y cells against 6-hydroxydopamine (6-OHDA)-induced oxidative stress and reduced the expression of pro-inflammatory mediators, including TNF-α, IL-1β, and IL-8, supporting its potential neuroprotective and anti-inflammatory effects (Latchoumycandane et al., 2011; Munni et al., 2023).

## 6 Molecular and cellular mechanisms of garlic in neuropathic pain

Garlic (*Allium sativum*) contains a diverse array of bioactive compounds with biological activities that may be relevant to the pathophysiology of NP. Approximately 184 secondary metabolites have been identified in garlic extracts, with several compounds reported to possess biological activities relevant to neuronal protection and pain-associated pathways (Rangarajan et al., 2021; Mahalati et al., 2025). Experimental studies suggest that garlic-derived compounds can modulate molecular pathways associated with oxidative stress, inflammation, neuronal survival, and nociceptive signaling (Shang et al., 2019; Tesfaye, 2021). Among the available evidence, DADS and DATS have demonstrated effects in chronic constriction injury (CCI) models, including attenuation of pain-related responses associated with modulation of the H₂S–BDNF–Nrf2 signaling pathway (Wang et al., 2020). However, the strength of evidence linking garlic-derived bioactives to NP varies substantially among experimental models and mechanisms (Koca et al., 2020; Savairam et al., 2023). A considerable proportion of the reported neuroprotective evidence has been generated using models of cerebral or spinal ischemia–reperfusion, 6-hydroxydopamine (6-OHDA)-induced Parkinsonian neurodegeneration, traumatic neural injury, and other forms of neuronal stress rather than established NP models (Grünblatt et al., 2000). These studies provide important supportive evidence for the antioxidant, anti-inflammatory, anti-apoptotic, and mitochondrial properties of garlic constituents, but they should not be interpreted as direct evidence of analgesic efficacy in NP. In contrast, studies conducted in nerve-injury models, particularly CCI models investigating DADS and DATS, provide more direct evidence for the potential relevance of garlic-derived OSCs to NP. Accordingly, the mechanisms discussed below distinguish direct NP-specific evidence from supportive evidence obtained from non-NP neurological models. This distinction is important when assessing the therapeutic relevance of garlic-derived bioactives, particularly in the absence of established human clinical evidence for their efficacy in NP (Figure 1).

**Figure 1:**
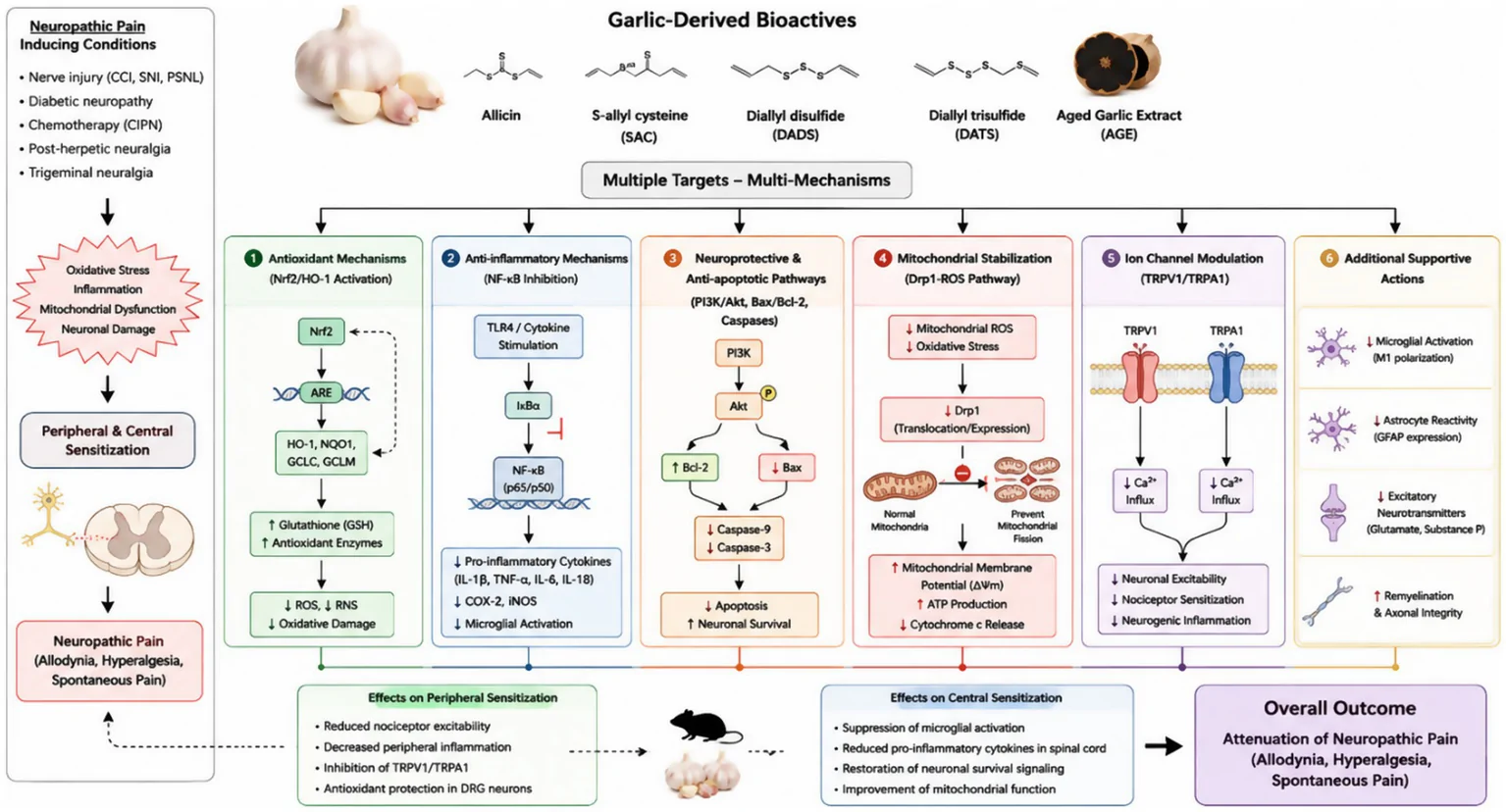
Overview of the proposed molecular mechanisms underlying the effects of garlic-derived bioactives in NP.

### 6.1 Antioxidant mechanisms

Oxidative stress is an important contributor to the development and persistence of NP particularly following peripheral nerve injury, where excessive ROS can interact with impaired endogenous antioxidant defenses and promote neuronal dysfunction and sensitization. Garlic contains a range of OSCs with antioxidant properties, influence cellular redox homeostasis by reducing oxidative burden and enhancing endogenous antioxidant defenses (Savairam et al., 2023). These activities are mechanistically relevant to NP because excessive ROS can damage lipids, proteins, nucleic acids, and mitochondrial components involved in neuronal homeostasis. Among garlic-derived compounds, allicin has been extensively investigated for its redox-modulating properties (Piriyanga et al., 2025). Allicin contains a highly reactive sulfur functionality that can interact with protein thiol groups and modify redox-sensitive cellular targets (Li et al., 2026; Sivalingam, 2026). Experimental studies have reported that allicin can scavenge or modulate reactive species, including superoxide, hydroxyl radicals, and peroxynitrite, thereby potentially limiting oxidative damage Allicin has also been associated with modulation of cellular antioxidant signaling, including activation of the nuclear factor erythroid 2-related factor 2 (Nrf2) pathway and increased expression of downstream cytoprotective enzymes such as heme oxygenase-1 (HO-1) (Yang et al., 2017; Meng et al., 2025). Similarly, AGE and its constituent fractions have been reported to influence Nrf2/HO-1-associated antioxidant responses and reduce oxidative and inflammatory stress in experimental neuronal and vascular models (Mathew and Biju, 2008; Hiramatsu et al., 2016). Activation of Nrf2 promotes transcription of antioxidant and cytoprotective genes and may therefore contribute to maintenance of neuronal redox homeostasis.

Garlic-derived compounds may additionally strengthen endogenous antioxidant defenses by increasing the activity or availability of superoxide dismutase (SOD), catalase (CAT), glutathione peroxidase (GPx), and glutathione (GSH) (Borek, 2004; Ahmed and Wang, 2021). GSH is particularly relevant to neuronal protection because it contributes to cellular redox regulation and protection against oxidative damage. Garlic constituents may also suppress pro-oxidant processes, including xanthine oxidase-dependent ROS generation, thereby providing an additional mechanism for limiting oxidative stress (Savairam et al., 2023). These effects collectively indicate that garlic-derived OSCs can influence both ROS generation and endogenous antioxidant capacity rather than acting solely as direct free-radical scavengers.

However, the strength of evidence linking these antioxidant effects specifically to NP requires careful consideration. A substantial proportion of the available evidence for allicin, AGE, SAC, and related garlic preparations has been obtained from cellular models and neurological injury or neurodegenerative models rather than from established NP models. Therefore, antioxidant signaling should be considered one component of a broader multimodal mechanism rather than an independently established analgesic mechanism of garlic in NP. Accordingly, findings from non-NP models should be regarded as supportive mechanistic evidence rather than direct evidence of therapeutic effectiveness against NP (Chen et al., 2014). Studies using CCI and other NP paradigms indicate that modulation of oxidative-stress pathways can be associated with attenuation of hyperalgesia and neuronal dysfunction (Abbaszadeh et al., 2018). Nevertheless, the NP-specific evidence for individual garlic constituents remains comparatively limited, with stronger direct evidence currently available for selected OSCs such as DADS and DATS than for garlic preparations or isolated compounds evaluated primarily in non-NP models (El-Saadony et al., 2024; Wu et al., 2026). Therefore, antioxidant signaling should be considered one component of a broader multimodal mechanism rather than an independently established analgesic mechanism of garlic in NP.

Overall, garlic-derived OSCs show clear potential to reduce oxidative stress by lowering ROS levels, activating the Nrf2/HO-1 pathway, and boosting the body’s natural antioxidant defenses. These mechanisms are relevant to NP, but most of the supporting evidence comes from non-NP models. This means antioxidant activity alone should not be assumed to translate into pain relief. Future studies using established NP models such as diabetic neuropathy, chemotherapy-induced neuropathy, and nerve-injury models are needed to confirm whether these antioxidant effects actually produce measurable analgesic benefits.

### 6.2 Anti-inflammatory mechanisms

Neuroinflammation is a key contributor to the development and persistence of NP, involving activation of immune and glial cells and increased production of proinflammatory mediators within the peripheral and central nervous systems. Studies that link changes in oxidative-stress pathways to actual pain outcomes in nerve-injury models should be given more weight. Several OSCs inhibit nuclear factor Kappa B (NF-Kb) a central regulator of inflammatory gene expression, thereby reducing the production of pro-inflammatory mediators such as nitric oxide (NO), prostaglandins, and cytokines (Mirzavandi et al., 2020). Meta-analytical evidence has also indicated that garlic supplementation can reduce circulating inflammatory markers, including C-reactive protein (CRP) and tumor TNF-α levels. In additionally, AGE has been shown to inhibit microglial activation and decrease IL-1β and TNF-α levels in brain regions such as the cerebral cortex and hippocampus (Nillert et al., 2017). These findings support the anti-inflammatory and neuroimmune-modulatory properties of garlic, although evidence obtained from systemic or non-neuropathic models should be distinguished from direct evidence in NP (Figure 2).

**Figure 2:**
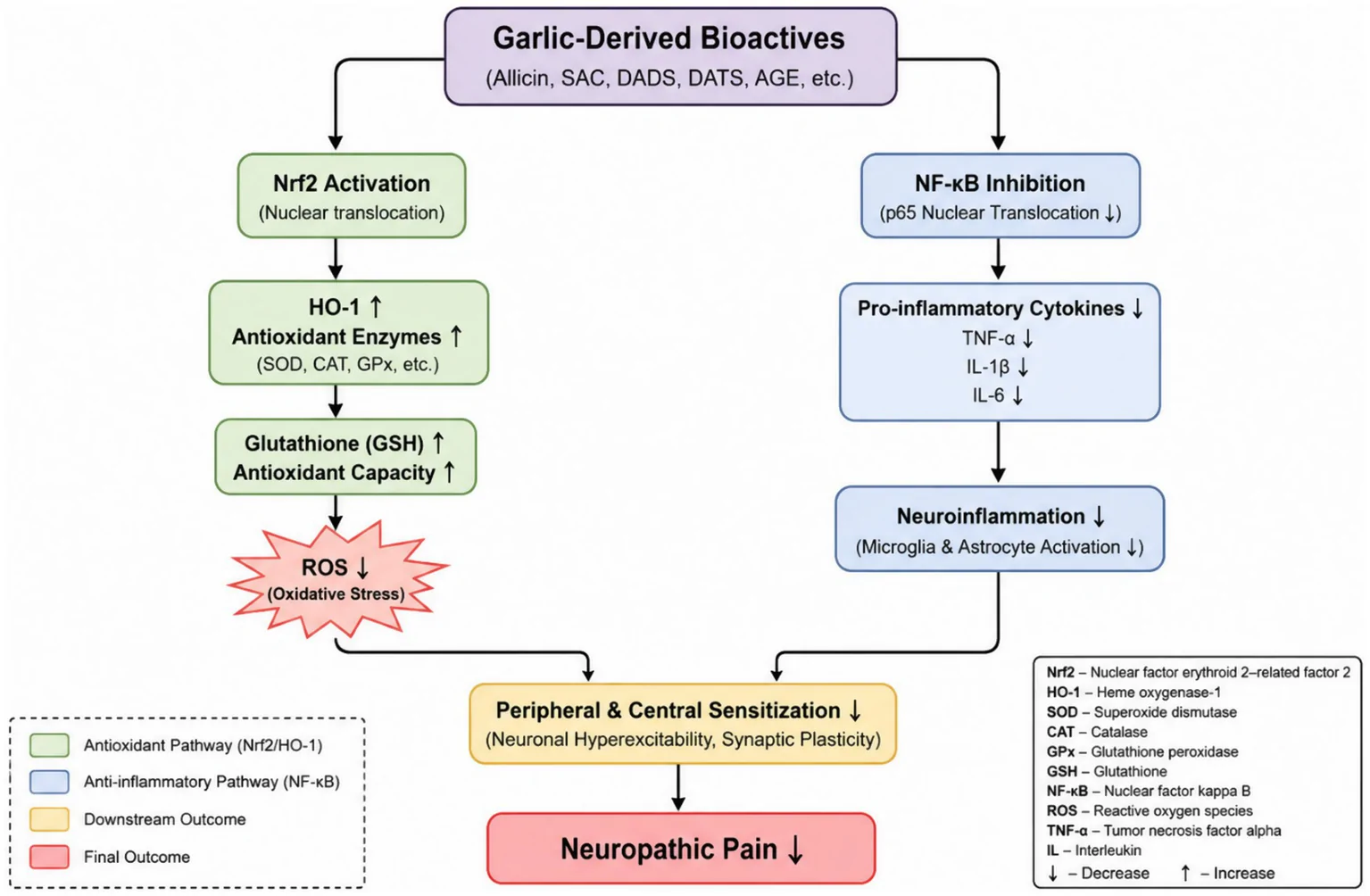
Antioxidant and anti-inflammatory mechanisms of garlic-derived bioactives in NP.

Several garlic-derived compounds, particularly allicin, have demonstrated anti-inflammatory and neuroprotective effects in experimental models of spinal cord injury (SCI), inflammatory injury, and cerebral ischemia–reperfusion. Allicin has been reported to reduce TNF-α, IL-1β, IL-6, and IL-8, and in cerebral ischemia reperfusion models, to attenuate tissue injury and neuronal apoptosis association with modulating TNF-α levels and myeloperoxidase activity (Gu and MacDermott, 1997). Protective effects have also associated with activation of the Nrf2/HO-1 signaling pathway (Li et al., 2012). Moreover, allicin has been reported to reduce transforming growth factor-beta (TGF-β) and CD68 expression and inhibits inducible nitric oxide synthase (iNOS), thereby limiting inflammatory responses (Sánchez-Gloria et al., 2021). SAC plays a complementary effects by regulating nitric oxide homeostasis and suppression iNOS expression (Mehrzia et al., 2006).

Garlic-derived compounds have also been reported to modulate inflammatory mediator release from activated glial cells, including microglia and astrocytes, which are important contributors to central sensitization (Zhou et al., 2014; Song et al., 2020). Lipophilic constituents such as DADS and DATS have likewise demonstrated anti-inflammatory properties (Song et al., 2016; El-Saadony et al., 2024). Importantly, most of the studies cited in this paragraph were performed in non-neuropathic inflammatory or neurological injury models. Therefore, these findings provide supportive mechanistic evidence for the anti-inflammatory potential of garlic-derived compounds but should not be interpreted as direct evidence of analgesic efficacy in NP.

Additional evidence from cellular inflammatory models further supports the ability of garlic preparations to regulate innate inflammatory signaling. Garlic extracts have been reported to inhibit (LPS)-induced Toll-like receptor 4 (TLR4) dimerization, and downstream NF-κB activation, accompanied by reducing COX-2 and iNOS expression. In RAW 264.7 macrophages, garlic extracts significantly decrease LPS-induced cytokine production and NO release while increasing HO-1 expression. Furthermore, garlic extracts inhibit the release of pro-inflammatory cytokines such as TNF-α, IL-1α, IL-6, and interferon-γ from leukocytes (Jeong et al., 2016; Moutia et al., 2018). These cellular findings establish the capacity of garlic constituents to interfere with inflammatory signaling but do not by themselves demonstrate an effect on nerve-injury-induced pain.

Taken together, garlic-derived bioactives, including allicin, SAC, DADS, DATS, and related sulfur-containing constituents, exhibit multi-targeted anti-inflammatory activity involving NF-κB, iNOS, COX-2, TLR4, cytokine signaling, and glial activation (Azam et al., 2025). Because these pathways contribute to peripheral and central sensitization, their modulation provides a plausible mechanistic basis for investigating garlic-derived compounds in NP (Cardelle et al., 2010; Arreola et al., 2015). Findings from systemic inflammatory, macrophage, ischemia–reperfusion, SCI, and other non-neuropathic models constitute supportive mechanistic evidence, whereas studies in validated nerve-injury models that demonstrate both suppression of neuroinflammatory signaling and improvement of pain-related behavioral outcomes provide stronger evidence for NP relevance (Bellanti, 2016). Thus, the anti-inflammatory properties of garlic-derived bioactives should currently be regarded as a promising mechanistic component of their potential activity against NP rather than as established clinical evidence of analgesic efficacy.

### 6.3 Neuroprotective and anti-apoptotic pathways

Garlic-derived OSCs have demonstrated neuroprotective and anti-apoptotic activities through modulation of multiple cellular survival and apoptotic pathways. These effects include regulation of the intrinsic mitochondrial apoptotic pathway and modulation of survival-associated signaling such as phosphoinositide 3-kinase/protein kinase B (PI3K/Akt) and c-Jun N-terminal kinase (JNK) (Lund et al., 2005). However, the strength of evidence linking these effects specifically to NP varies considerably among experimental models. The available evidence has been obtained from neuronal oxidative-stress, neurodegenerative, ischemia–reperfusion, or traumatic neural injury models rather than established nerve injury models of NP (Olufunmilayo et al., 2023). Therefore, these findings provide supportive evidence for the neuroprotective properties of garlic bioactive, but should not be interpreted directly as evidence of analgesic efficacy in NP.

A major component of the intrinsic apoptotic pathway is the balance between pro-apoptotic Bcl-2-associated X protein (Bax) and the anti-apoptotic protein B-cell lymphoma 2 (Bcl-2). Under pathological conditions, an increased Bax/Bcl-2 ratio promotes mitochondrial membrane permeabilization, facilitating cytochrome c release and subsequent activation of downstream caspases (Kroemer et al., 2007). Garlic-derived compounds, particularly S-allyl cysteine (SAC), have been reported to influence this apoptotic balance and thereby support neuronal survival. However, evidence for these effects is largely derived from experimental models of neuronal injury or neurodegeneration. Thus, while regulation of the Bax/Bcl-2 axis provides a biologically plausible mechanism through which garlic compounds could protect neurons following pathological stress, its specific contribution to the attenuation of NP remains insufficiently established.

Garlic OSCs may additionally influence intracellular survival and stress-response pathways. SAC and DADS have been associated with modulation of PI3K/Akt and JNK signaling, pathways that regulate cell survival, mitochondrial integrity, and stress-induced apoptosis (Yao et al., 2005). In nerve growth factor-differentiated PC12 cells exposed to oxidative stress, DADS has been reported to enhance cell viability through activation of the PI3K/Akt pathway and inhibition of glycogen synthase kinase-3 (GSK-3). These effects were accompanied by reduced cytochrome c release, caspase-3 activation, and poly (ADP-ribose) polymerase (PARP) cleavage, indicating suppression of apoptosis (Zhang et al., 2015; Farooqui and Farooqui, 2018). Although these findings provide evidence for the cytoprotective effects of DADS under neuronal oxidative stress, the PC12 model does not reproduce the complex pathological features of nerve-injury-induced NP.

Allicin has also been demonstrated anti-apoptotic and neuroprotective effects in experimental ischemia–reperfusion and traumatic brain injury models. These effects have been associated with attenuation of oxidative stress, lipid peroxidation, and modulating PI3K/Akt and NF-κB signaling pathways (Lee et al., 2018). Similarly, garlic supplementation has been reported to reduce neuronal apoptosis and improved neurological outcomes in experimental ischemia–reperfusion injury (Pari and Murugavel, 2007). Z-ajoene and SAC have also demonstrated protective effects on hippocampal neurons, including reducing in lipid peroxidation, gliosis, and delayed neuronal death (Yoo et al., 2014). Collectively, these studies support the broader neuroprotective capacity of garlic-derived compounds; however, because they were conducted primarily in ischemic, traumatic, or neurodegenerative settings, they should not be considered as direct evidence that these compounds prevent or reverse NP.

From an NP perspective, the distinction between neuronal protection and analgesic efficacy is particularly important. NP involves not only neuronal injury or apoptosis but also persistent peripheral and central sensitization, abnormal sensory signaling, neuroinflammation, and altered neuronal excitability (Martins et al., 2026). Therefore, suppression of apoptosis in a neuronal injury model does not necessarily predict an improvement in NP behavior (Hamdan et al., 2024). Evidence obtained from validated nerve-injury models, such as CCI, is consequently more directly relevant when evaluating the therapeutic significance of garlic-derived bioactives in NP. The currently available NP-specific evidence is comparatively limited, with DADS and DATS providing more direct experimental support through studies in CCI models (El-Saadony et al., 2024). These findings suggest that garlic OSCs may influence nerve-injury-associated pain mechanisms, but the relatively small number of NP-specific studies prevents definitive conclusions regarding their therapeutic efficacy.

Therefore, the anti-apoptotic actions of garlic-derived compounds should currently be interpreted as one component of a broader multitarget mechanism involving oxidative stress, inflammation, neuronal survival signaling, and ion-channel regulation. Evidence from non-NP neurological models strengthens the biological rationale for these effects but should be distinguished from direct evidence obtained in NP models. Further studies using disease-relevant NP models are required to determine whether modulation of Bax/Bcl-2, caspase activation, PI3K/Akt, JNK, and related survival pathways translates into sustained reductions in NP behavior. The absence of well-designed human clinical studies specifically evaluating these mechanisms in NP also warrants caution in therapeutic interpretation (Figure 3).

**Figure 3:**
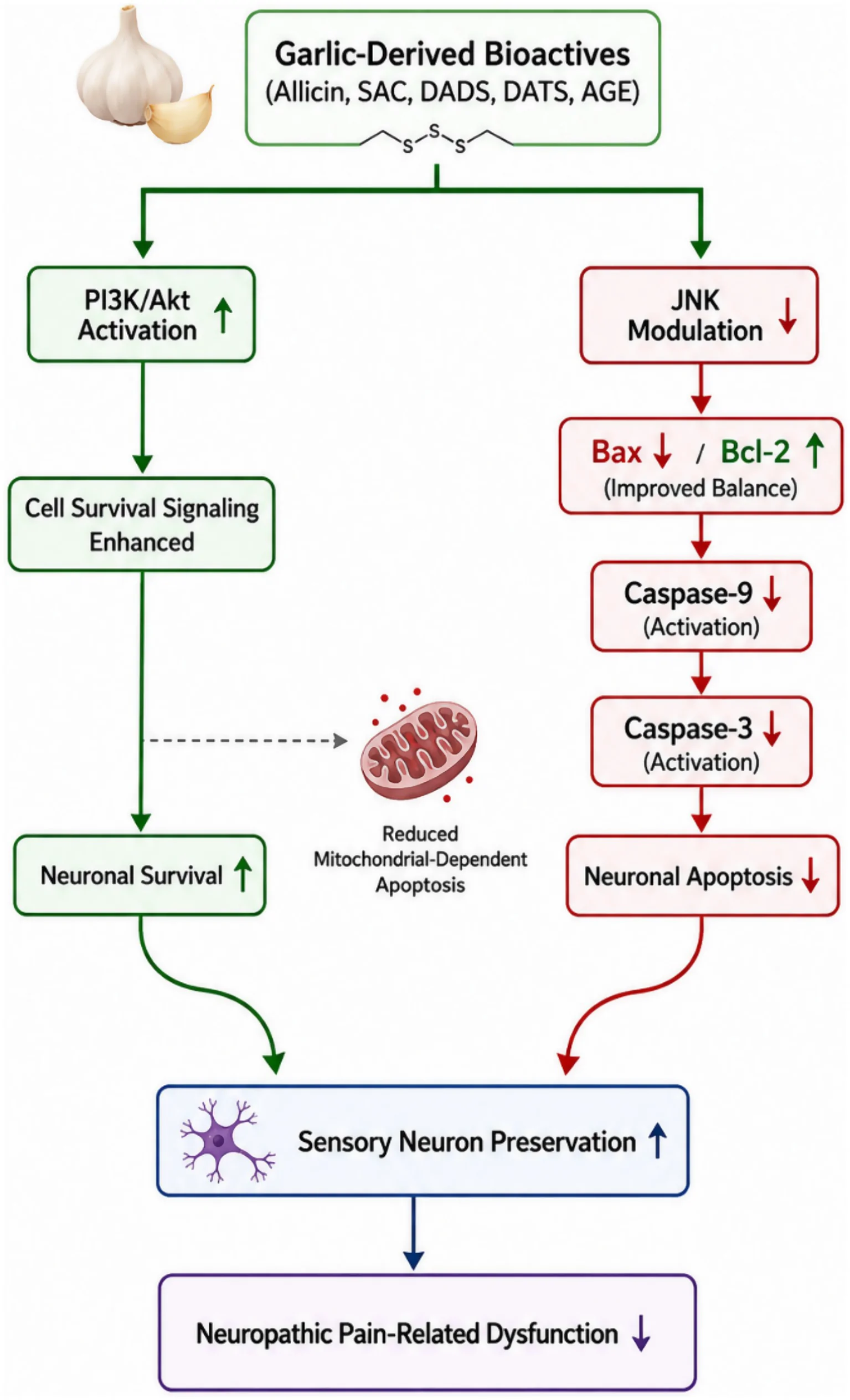
Neuroprotective and anti-apoptotic signaling pathways modulated by garlic-derived bioactives in NP.

### 6.4 Modulation of ion channels (TRPV1/TRPA1)

Modulation of transient receptor potential (TRP), particularly TRPV1 and TRPA1, represents an important mechanism involved nociceptive signaling and neuronal sensitization in NP. However, although TRPV1/TRPA1 are established pain-related molecular targets, evidence directly demonstrating that garlic-derived bioactive alleviate NP through these channels remains comparatively limited. Allicin activates TRPA1 and has also been reported to interact with TRPV1, although its activity at TRPV1 is relatively weak than that of well-established principal agonists such as capsaicin. Allicin, an unstable component of fresh garlic, is the chemical responsible for TRPA1 and TRPV1 activation and is therefore likely to cause garlic’s pungency (Macpherson et al., 2005). However, Bautista et al. (2005) showed that allicin and DADS excite an allyl isothiocyanate-sensitive subpopulation of sensory neurons and induce vasodilation by activating capsaicin-sensitive perivascular sensory nerve endings, suggesting that garlic excites sensory neurons primarily through activation of TRPA1 rather than TRPV1. This is consistent with subsequent characterizations of TRPA1 as the channel activated by a range of diverse stimuli, including cold temperature, mechanical stress, and pungent compounds such as AITC, allicin, and cinnamaldehyde (Story et al., 2003; Bandell et al., 2004), reinforcing that TRPA1, not TRPV1, is allicin’s primary and functionally dominant target. TRPV1 involvement, where reported, is comparatively minor and secondary to this main action (Macpherson et al., 2005).

Experimental studies have demonstrated that allicin activated populations of sensory neurons that are sensitive to capsaicin and cinnamaldehyde, indicating its ability to modulate TRPV1 and TRPA1 associated signaling (Song et al., 2020; Khan et al., 2022). The interaction of allicin with TRPV1 is therefore mechanistically relevant to sensory signaling; however, this effect should not be interpreted as inherently analgesic because acute TRPV1 activation by allicin can itself evoke nociceptive responses. Consequently, any potential therapeutic relevance would depend on factors such as exposure level, duration, repeated stimulation, and subsequent changes in channel sensitivity rather than on simple channel activation alone (Amagase, 2006). Experimental studies have suggested that repeated or sustained stimulation of TRPV1 can produce desensitization of sensory neurons and attenuation of subsequent nociceptive responses (Caterina et al., 1997; Negri et al., 2006). This phenomenon provides a possible mechanistic basis for considering TRPV1 modulation in pain control; however, direct evidence that garlic-derived allicin produces clinically meaningful TRPV1 desensitization in NP is currently insufficient. Similarly, although TRPV1 is involved in trigeminal nociception, the available evidence does not establish garlic-derived bioactives as effective treatments for trigeminal neuralgia or post-herpetic neuralgia. These disorders should therefore be considered potential areas for future investigation rather than established therapeutic indications (Macpherson et al., 2005; Vijayan et al., 2025). Previous study showed that in complete Freund’s adjuvant induced chronic inflammatory pain in rate, the TRPA1 and TRPV1 activity at the spinal level contributes to enhance excitatory nociceptive input from primary sensory nerves to dorsal horn neurons (Huang et al., 2019). The mouse trigeminal NP model showed increased afferent signaling and mechanical hyperalgesia by TRPV1 sensitization at the central terminal of primary sensory neurons (Kim et al., 2014).

Guo et al. (2019) demonstrated that intrathecal TRPV1 gene silencing attenuated mechanical and thermal hyperalgesia and reduced CaMKII expression and ERK2 phosphorylation in the spinal cord of rats subjected to CCI, directly linking spinal TRPV1 upregulation to the maintenance of NP. Similarly, Miyakawa et al. (2014) using *in vivo* patch-clamp recordings in a nerve-root constriction rat model, showed that TRPA1 antagonism reduced excitatory synaptic transmission in substantia gelatinosa neurons of the spinal dorsal horn, providing direct electrophysiological evidence for TRPA1’s role in central pain transmission. Additional support for central/glial involvement comes from studies showing TRPA1 expression in spinal astrocytes and its contribution to neuroinflammation-associated pain hypersensitivity in mouse models of demyelinating disease. These *in vivo* and neuronal-level studies offer more direct and mechanistically relevant evidence for TRPV1/TRPA1 involvement in central sensitization.

TRPA1 is another important chemosensory channel associated with NP. It is activated by reactive electrophiles and endogenous products of oxidative stress and contributes to mechanical and cold hypersensitivity in several experimental pain conditions. Garlic-derived OSCs may interact with TRPA1-related signaling because of their electrophilic and redox-sensitive properties. In addition, studies using chronic constriction injury (CCI) models have indicated that DADS and DATS can attenuate NP-related responses while modulating H₂S-associated signaling pathways (Macpherson et al., 2005; Wang et al., 2020). These findings provide comparatively stronger NP-specific evidence than from non-neuropathic neuronal models. Nevertheless, the available CCI studies do not establish that direct TRPA1 modulation is the principal mechanism responsible for the observed analgesic effects of DADS or DATS; therefore, this relationship should currently be regarded as mechanistically plausible rather than definitively demonstrated. The relevance of TRPV1 and TRPA1 to diabetic neuropathy and chemotherapy-induced peripheral neuropathy (CIPN) further supports their consideration as potential molecular targets because abnormal TRP-channel activity can contribute to peripheral sensitization, spontaneous pain, and sensory hypersensitivity in these conditions (Koizumi et al., 2009; Iannone et al., 2023).

However, the presence of TRP-channel dysregulation in these neuropathic conditions does not establish that garlic-derived compounds can therapeutically normalize these channels. Disease-specific studies are therefore required to determine whether individual garlic bioactives, particularly DADS, DATS, and allicin, modify TRPV1/TRPA1 activity in diabetic neuropathy, CIPN, post-herpetic neuralgia, or other clinically relevant NP models (Bautista et al., 2005; Hernández-Cruz et al., 2022). Overall, garlic-derived bioactives demonstrate the capacity to interact with TRPV1- and TRPA1-associated sensory pathways, providing a plausible molecular link between garlic constituents and nociceptive signaling. However, the current evidence should be interpreted cautiously. Much of the mechanistic evidence for TRP-channel modulation is derived from sensory-neuron or general nociceptive studies, whereas direct evidence in established NP models remains limited. The available CCI evidence involving DADS and DATS provides more direct support for NP relevance, but the specific contribution of TRPV1/TRPA1 to these effects remains to be established. Furthermore, the absence of convincing human clinical studies directly evaluating garlic-derived compounds as TRPV1/TRPA1-modulating interventions in NP precludes definitive therapeutic conclusions. Thus, TRPV1/TRPA1 modulation should presently be considered a promising mechanistic hypothesis rather than an established therapeutic mechanism of garlic in NP (Figure 4).

**Figure 4:**
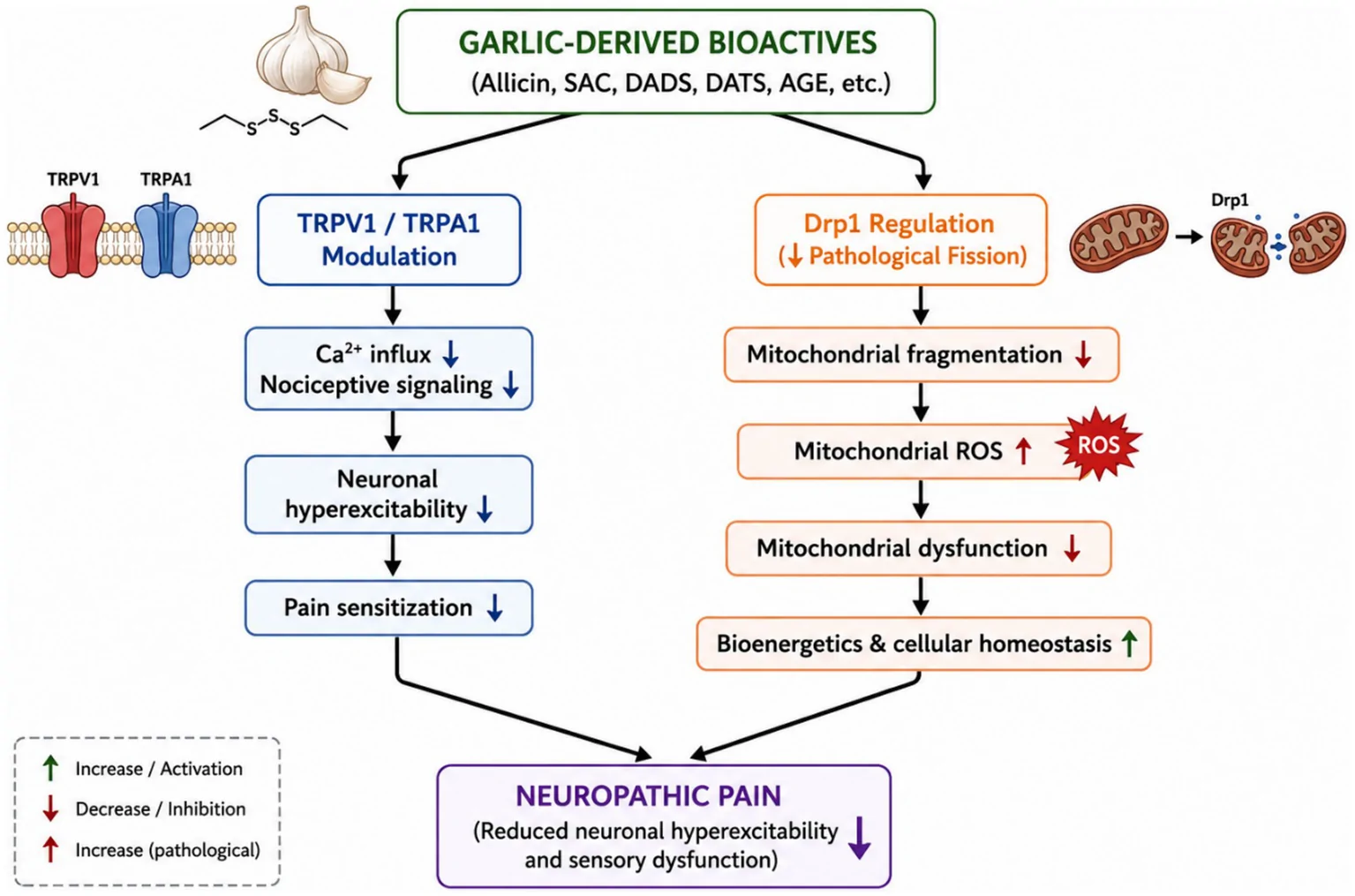
Modulation of nociceptive ion channels and mitochondrial dysfunction by garlic-derived bioactives in NP.

### 6.5 Mitochondrial stabilization

Mitochondrial dysfunction is an important component of NP pathophysiology, particularly in conditions such as diabetic neuropathy and CIPN. Impaired mitochondria bioenergetics, excessive mitochondrial ROS generation, altered mitochondrial dynamics, and disruption of neuronal energy homeostasis can contribute to sensory neuronal dysfunction and persistent pain (Mathew and Biju, 2008; Mirzavandi et al., 2020). However, the mitochondrial abnormalities associated with NP have been discussed in previously in Section 4.1; therefore, this section focuses specifically on the evidence linking garlic-derived bioactives with mitochondrial regulation.

Among garlic-derived OSCs, DADS and DATS provide comparatively stronger evidence directly relevant to NP. Experimental studies using CCI models have reported that DADS and DATS attenuate NP-related responses and modulate signaling associated with oxidative and neuroprotective pathways. These findings are particularly relevant because they were obtained in a nerve-injury model of NP rather than in a general neurodegenerative or neuronal injury model (Wang et al., 2020; Hernández-Cruz et al., 2022). Nevertheless, the available NP-specific evidence remains limited, and the contribution of mitochondrial stabilization to the observed analgesic effects of DADS and DATS has not been established as an independent mechanism. Their effects may involve interconnected antioxidant, neurotrophic, and inflammatory pathways, including the H₂S–BDNF–Nrf2 axis. Therefore, mitochondrial modulation should currently be considered one possible component of the broader actions of these compounds rather than a definitively established mechanism of garlic-mediated analgesia in NP (Yeni et al., 2024).

Garlic-derived compounds may influence mitochondrial homeostasis by reducing oxidative and nitrosative stress, preserving mitochondrial integrity, and supporting cellular antioxidant defenses. SAC, for example, has demonstrated neuroprotective activity in experimental models of neuronal injury. The 6-hydroxydopamine (6-OHDA)-induced Parkinsonian model, SAC improved neuronal viability and reduced oxidative and inflammatory responses, suggesting a capacity to protect neurons under conditions of oxidative stress (Latchoumycandane et al., 2011). However, this model represents Parkinsonian neurodegeneration rather than NP. Accordingly, these findings provide supportive evidence for the mitochondrial and neuroprotective properties of SAC but should not be interpreted as direct evidence of efficacy against NP.

Similarly, AGE and its fractions have demonstrated mitochondrial and antioxidant effects in non-neuropathic experimental systems. The ethyl acetate fraction of AGE reduced intracellular ROS generation and lipid peroxidation in β-amyloid-stimulated PC12 cells, thereby improving cell viability and attenuating neurotoxicity (Jeong et al., 2013; Kanamori et al., 2020). Other studies have reported protective effects of SAC and AGE in ischemia–reperfusion models, including preservation of glutathione (GSH), inhibition of lipid peroxidation, maintenance of mitochondrial function, and preservation of mitochondrial membrane potential and ATP production. These findings support the broader neuroprotective and mitochondrial effects of garlic preparations; however, because these experiments were conducted in ischemic or neurodegenerative models rather than established NP models, they should be regarded as indirect mechanistic evidence.

The potential relevance of mitochondrial protection is particularly important in diabetic neuropathy. Persistent hyperglycemia can increase mitochondrial ROS generation, impair ATP production, and disrupt mitochondrial dynamics in sensory neurons, thereby contributing to neuronal dysfunction. Similarly, chemotherapeutic agents used in cancer treatment can induce mitochondrial stress, mitochondrial DNA damage, altered mitochondrial dynamics, and impaired axonal function, processes implicated in CIPN (Doyle and Salvemini, 2021). These observations establish mitochondrial dysfunction as a relevant therapeutic target in diabetic neuropathy and CIPN. However, direct evidence demonstrating that garlic-derived bioactive reverse these mitochondrial abnormalities and consequently reduce diabetic or chemotherapy-induced NP remains limited. Thus, the potential application of garlic-derived compounds in these conditions should be considered mechanistically plausible but not yet clinically established (Sener et al., 2007).

The mitochondrial effects of garlic-derived compounds may also depend on the cellular context, compound concentration, and disease state. For example, DADS has been reported to induce mitochondrial depolarization in tumor cells, indicating that its effects on mitochondrial function are not uniformly protective across all biological systems (Nasr, 2014; Kanamori et al., 2020). This context dependence highlights the importance of distinguishing antioxidant or neuroprotective effects observed under specific experimental conditions from generalized claims of mitochondrial stabilization. It also emphasizes the need to establish dose-dependent effects and tissue-specific responses before therapeutic translation (Khacho et al., 2017).

Another important consideration is the substantial heterogeneity among garlic preparations. Fresh garlic, AGE, black garlic, and preparations enriched in individual OSCs differ considerably in their chemical composition, stability, bioavailability, and relative concentrations of compounds such as allicin, SAC, DADS, and related sulfur-containing metabolites (Hernández-Cruz et al., 2022; Utama et al., 2024). Consequently, mitochondrial effects observed with purified OSCs cannot automatically be extrapolated to whole-garlic preparations, and findings obtained with AGE or black garlic cannot necessarily be generalized to fresh garlic. Standardization of the chemical composition and characterization of bioactive constituents will therefore be essential for reproducibility, dose optimization, and comparison among future studies.

Despite promising preclinical findings, human evidence directly evaluating garlic-derived bioactives for NP remains insufficient. In particular, there is currently a lack of well-designed clinical studies demonstrating that garlic preparations or individual OSCs improve NP through mitochondrial mechanisms. Important translational questions concerning pharmacokinetics, tissue exposure, bioavailability, optimal dosing, formulation stability, and long-term safety also remain unresolved (Mathew and Biju, 2008; Ahmed and Wang, 2021). Therefore, evidence from ischemia reperfusion, Parkinsonian, β-amyloid, and other neurodegenerative models should be interpreted as supportive mechanistic evidence rather than as clinical or direct NP evidence.

Overall, mitochondrial stabilization represents a biologically relevant but incompletely validated mechanism through which garlic-derived bioactives may influence NP. The strongest evidence currently available for NP relevance is derived from a limited number of nerve-injury studies involving DADS and DATS, whereas much of the evidence for SAC, AGE, and other garlic preparations originates from non-NP models of neuronal oxidative stress and neurodegeneration. Future studies should therefore prioritize validated models of diabetic neuropathy, CIPN, and other clinically relevant NP conditions and should simultaneously evaluate pain-related behavioral outcomes with mitochondrial endpoints, including mitochondrial ROS, membrane potential, ATP production, mitochondrial dynamics, and Drp1-associated signaling. Well-designed human clinical trials using chemically standardized garlic preparations or defined OSCs will ultimately be required to determine whether these mitochondrial effects translate into clinically meaningful benefits. At present, garlic-derived bioactives should therefore be considered potential adjunctive candidates rather than established mitochondrial-targeted therapies for NP.

## 7 Limitations and future directions

Despite promising preclinical evidence, several limitations hinder the clinical translation of garlic in NP management. A major challenge is the substantial variability in garlic preparations, including fresh garlic, AGE, and black garlic, which differ in their chemical composition and bioactive profiles. This heterogeneity results in inconsistent concentrations of key OSGs such as allicin and SAC, making standardization and dose optimization difficult. For instance, SAC is enriched in AGE, whereas black garlic contains distinct sulfur compounds, including dithiol-thiones and trithiolanes, which may exert unique mitochondrial effects (Kanamori et al., 2020; Ahmed and Wang, 2021; Yeni et al., 2024).

Another significant limitation is the paucity of well-designed clinical trials, as most current evidence is derived from *in vitro* and animal studies. The bioavailability, pharmacokinetics, and optimal therapeutic dosing of garlic-derived compounds remain insufficiently characterized. In addition, the instability of certain constituents, particularly allicin, poses challenges for formulation and delivery (Sasmaz et al., 2025). Potential interactions with conventional medications and the risk of dose-dependent adverse effects further necessitate careful evaluation before clinical application.

Future research should focus on the development of standardized garlic formulations with well-defined bioactive profiles, along with rigorous clinical trials to establish efficacy and safety in human NP conditions. Advanced delivery systems aimed at improving stability and bioavailability, as well as studies elucidating pharmacokinetics and molecular targets in humans, are essential. Given their multitargeted mechanisms modulating oxidative stress, inflammation, ion channels, apoptosis, and mitochondrial function garlic-derived compounds hold considerable promise as adjunctive therapies. When integrated with existing treatment strategies, they may enhance therapeutic outcomes, particularly in conditions such as diabetic neuropathy and CIPN.

## 8 Conclusion

It is evident that garlic-derived bioactive compounds exert significant neuroprotective effects, primarily through stabilization of mitochondrial function by preserving mitochondrial membrane potential, enhancing endogenous antioxidant defense, regulating mitochondrial dynamics, and supporting cellular energy metabolism. In addition, their multitargeted actions involving modulation of oxidative stress, neuroinflammation, apoptotic pathways, and ion channel activity further contribute to their therapeutic potential in NP. Although substantial preclinical evidence supports these beneficial effects, clinical validation remains limited. Therefore, further well-designed studies are required to confirm their efficacy, optimize dosing, and establish standardized formulations for effective application in NP management.
